# Hailey-Hailey disease: the role of azathioprine an immunomodulator

**DOI:** 10.11604/pamj.2019.32.65.17877

**Published:** 2019-02-05

**Authors:** Malumani Malan, Wu Xuejingzi, Jiang Si, Song Ji Quan

**Affiliations:** 1 Department of Dermatology and Venereology at Zhongnan Hospital of Wuhan University, Wuhan City, Hubei Province, China; 2 Livingstone Central Hospital, Southern Zambia

**Keywords:** Familial benign chronic pemphigus, Hailey-Hailey disease, azathioprine, benign chronic familial pemphigus, benign chronic pemphigus, familial benign pemphigus

## Abstract

Hailey-Hailey disease (HHD) is a rare autosomal dominant hereditary blistering and erosions disorder affecting the intertriginous regions of the body. There is still no treatment protocol for this disease thus clinicians are highly advised to draw up individualized treatment plan. In this case report, we discuss a case of HHD in a 58-year-old Chinese man who was successfully treated with azathioprine in Hubei province.

## Introduction

Hailey-Hailey disease (HHD), named after the two brothers Howard and Hugh Hailey who first described this still rare autosomal dominant hereditary blistering and erosious disorder affecting the intertriginous regions of the body in 1939 [1]. HHD is synonym as benign chronic familial pemphigus, benign chronic pemphigus or familial benign pemphigus [2]. It is not an autoimmune disorder and thus there are no autoantibodies. The first onset of HHD generally occurs between 20 years and 40 years of age. Xu *et al.* in 2017, approximate that the prevalence of HHD is 1: 50,000 worldwide [3, 4]. The onset of symptoms usually occurs around puberty or middle age and may be exacerbated by perspiration, weight gain, infection, trauma, pregnancy and ultraviolet radiation [5]. This rare disease is due to a heterozygous autosomal dominant mutation in the ATP2C1 gene [2] on chromosome 3, which encodes for secretory pathway of adenosine triphosphate-dependent calcium/manganese pump (Ca2+/Mn2+ ATP-ase protein (SPCA1)) in the Golgi apparatus whose function is to maintain intercellular calcium homeostasis thus leading to alterations in Ca2+-dependent intracellular signaling and resulting in the loss of cellular adhesion in the epidermis [6, 7]. Clinically patients with HHD presents with a long history of wax and wanes of symptoms; these include but not limited to the following dermatosis characterized by chronic, recurrent vesicles, erosions, and maceration in flexural areas friction areas, mainly in the axillae, submammary folds, groin, perineum and neck. However, lesions also can occur in non-skin-fold areas [8]. The other associated features are itchiness, burning sensation, pain and fever which are mainly complications of the primary pathology. Chiaravalloti and Payette, 2014 record that nail changes are part of a criteria to diagnose HHD. The duo documents that about 71% of patients have asymptomatic linear, white bands in the nail plates. According to them, nail changes has been the presenting feature in many cases [4, 5, 9]. In this case review, we shall discuss a case of HHD, review the commonest triggers and the role of azathioprine, an immunosuppressive agent in the management of the disease.

## Patient and observation

A 50-year-old Asian male was referred to our outpatient department from a local clinic with a diagnosis of familial benign pemphigus for 10 years and well controlled hypertension for 20 years now. With the current hospital visit, he had been complaining of waxing and waning lesions under armpit and groin which where dark-reddish in colour with many papular-vesicles which could easily break and leave erosions and ulcers in these regions and later on form scab. They were associated with lots of pain which was initially itchy and fever for the past five days’ prior presentation to our hospital. This presentation limited his normal daily chaos. He has not received much improvement from the usual topical and oral medication which he has been taking from since past the illness started.

**Figure 1:**
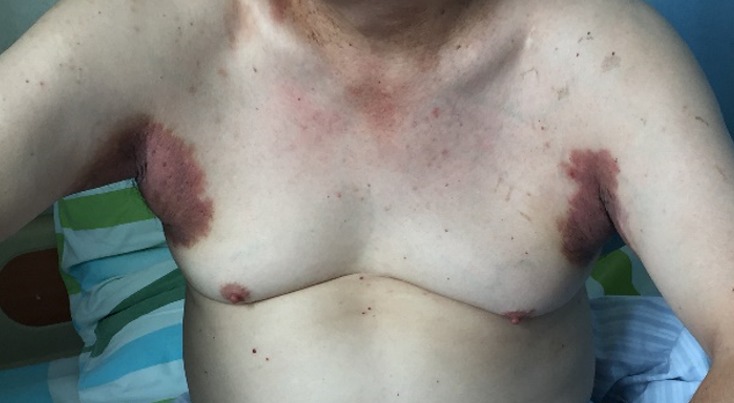
Bilateral axilla showing erythematous lesions with associated erosions, some fissuring, crusts, pigmentation and scale at friction sites in a patient with HHD

**Figure 2:**
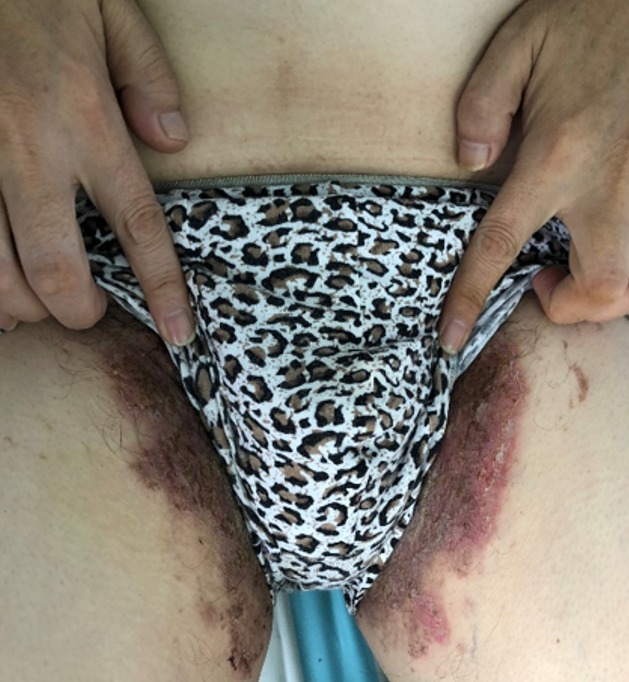
Bilateral inguinal region showing erythematous pigmentation of the lesions with associated crusts and scaling finger nails with faint longitudinal streaks of white bands

The review of other system was unremarkable. He does not have diabetes mellitus, endocrinological nor any autoimmune disorder. He is retroviral status non-reactive and neither does he have any infectious disease like tuberculosis, syphilis, hepatitis A, B, C. He has a positive family his of similar clinical presentation in the late mother. He was febrile to touch with axillary temperature of 38.0°C and other vitals where within normal range. Dermatological exam reviewed, diffuse edematous dark erythematous lesions seen in both axillaries and groins as shown in Figure 1 and Figure 2. Erythematous lesions had associated erosions, crusts, blisters, pigmentation, and scale at friction sites, accompanied by pain, itching and malodor. Erosive surfaces left by the ulceration of blisters to be seen with a small amount of exudation and scab on the surface. Nikolsky’s sign was positive.

The rest of the examination was unremarkable. He was treated as Hailey-Hailey disease in view of the positive family history in mother, characteristic lesions in suspect flexure areas, relapsing course, nature of the illness and the referring clinicians’ diagnosis. However, he did not show the classical asymptomatic linear, white bands in the nail plates as documented by many authors [9]. We did not do a biopsy and PCR for genetic mutation of ATP2C1 to confirm diagnosis due to financial constraints. All radiologic investigations were normal.

The patient was empirically initiated on both topical and systemic agents for disease modifying and symptomatic treatment: injectable glycyrrhizin acid, analgesic- codeine phosphate, anti-histamine; cetirizine for symptomatic relief and topical cefoperazone tazobactam in view of the fevers. Day 3 on this cocktail showed little improvement in disease improvement with blood test results suggesting that the disease is in active stage thus, an immunomodulator, azathioprine orally was added to the regimen. Other supporting drugs where directed by laboratory findings. For hypoalbuminemia, he was advised on a high-quality protein diet and fluid ingestion in view of the high uric levels.

Most supportive investigations done where unremarkable as indicated in Table 1. Of note was the raised markers of inflammation, erythrocyte sedimentation rate of 64mm/hour, positive skin exudation culture for *Streptococcus agalactis*, *monocytosis* of 15.6% monocyte absolute value: 1.15 x 10^9^/L. He also had hypokalemia and hypoalbuminemia and hypoglobuminemia. Laboratory biochemistry tests, complete blood count, serum viral screen, autoimmune screen; ANA+ENA, ANCA spectrum, GM antibody determination and rheumatoid factor, TORCH screen infections, potassium hydroxide scrapping for fungal, fungi 1-3-B-D dextran quantitative G test, Stool and urine analysis were unremarkable.

**Table 1:** Laboratory investigations done

Tests	Components	Value in SI units	Reference Range
Inflammatory Markers	ESR	64mm/hour	
	Procalcitonin	0.67ng/m1	
	Hypersensitive C reactive protein (HS-CRP:5)	1.14mg/L	0-5
	rheumatoid factor	< 11kU/L	
Biochemistry Tests	ALT	59U/L	9-50
	AST	58U/L	15-40
	GGT	59U/L	10-60
	superoxide dismutase	7.5U/L	
	creatine kinase	281U/L	50-310
	LDH	246U/L	120-250
	Vitamin C	628mg/dL	
	albumin (ALB)	36.4g/L	40-55
	globulin	31.9g/L	20-40
	uric acid	474.5mmol/ L	208-428
	sialic acid (SA)	1013.2mg/L	
	high density lipoprotein (HDL)	0.64mmo1/L	1.16-1.42
	Apolipoprotein A1 (ApoA1)	0.71g/L	1.2-1.6
	serum amyloid A	181.20mg/L	
	potassium (K +)	3.3mol/L	3.5-5.3

**Figure 3:**
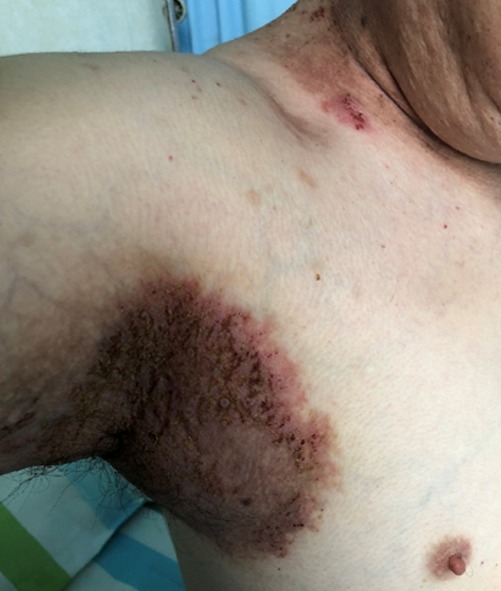
Day five post treatment with azathioprine, indicating healing erythematous axilla with some healing fissures, crustations and scaling

**Figure 4:**
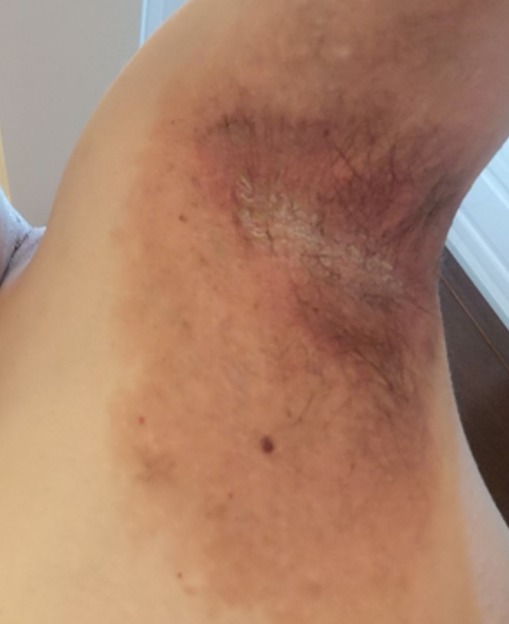
Left axilla of the HHD patient after 3 months on azathioprine. There is some post inflammatory pigmentation and crustation and scaling in the central region of axilla

The patient showed much improvement after day 5 on azathioprine and he was treated as outpatient on the same cocktail with a 2 weekly supply of the immunomodulator as indicated in Figure 3, Figure 4.

## Discussion

Management of genetically influenced diseases like HHD can always be very challenging because most of them rarely have a cure but they can be managed clinically as and when symptoms evolve. HHD is such a disease which requires patient education on their illness, its possible triggers or precipitants, how to both avoid and manage flares. They also require special education on the various kinds of drugs used for their condition. Our patient prior being referred to us, used a series of both topical and oral antibiotics which were becoming less effective for his condition. During our first contact, he was put on anti-allergic treatment (compound glycyrrhizin and calcium aspartate intravenously), topical antibiotic (cefoperazone tazobactam topical drug) for both symptomatic and disease modification treatment. Day three post admission, the disease was still active evidenced by laboratory and clinical evaluation and a consensus to add an immunosuppressive agent was reached, azathioprine was included on the drug list. The use of azathioprine in management of HHD has been reported although in combination with other drugs [10, 11].

For instance, dermatologists have been using azathioprine to successfully treat immunobullous diseases for nearly 35 years. Consequently, this application has become well recognized, and azathioprine has thus been ‘‘grandfathered’’ into the dermatologic armamentarium despite the lack of multiple prospective randomized, double-blinded controlled trials to support its use [12]. Symptoms are exacerbated by factors including sun exposure, heat, sweat and friction [5] as was with our patient. Burge S.M, 1992, notes that exacerbations have also been observed with patch testing and herpes simplex virus type 1, with possible evolution into herpetic eczema [13]. As for our client viral screen for herpes simplex virus type 1 was unsatisfactory. HHD is an autosomal dominant skin disorder characterized by abnormal keratinocyte adhesion in the suprabasal layer of the epidermis.

On histology, suprabasilar acantholysis is often described as “a row of tombstones” or a “dilapidated brick wall” - the “tombstones” or “bricks” referring to the basilar keratinocytes [4]. Dyskeratosis may be present, and the dermis is rarely affected, though perivascular lymphocytic infiltrate may be found [13]. Furthermore, unlike in bullous diseases such as pemphigus vulgaris, antidesmosomal autoantibodies might not be detected as described by Chiaravalloti and Payette, 2014 [4].

## Conclusion

Treatment of HHD should be individualized because of the rarity and nature of disease which is chronic and characterized by occasional spontaneous remissions and multiple recurrences. Our patient responded so well to azathioprine and thus, it adds to the list of case studies in the management of HHD. The drug can be listed on the potential drugs in the management of this rare disorder but in an individualized pattern taking care of possible adverse effects of the drug. The use of other immunomodulators has been documented by Arora *et al.*, 2016. Topical therapy should also be encouraged in view of the chronicity of the condition and patients should be advised to avoid precipitants at all cost and also adopt a conservative approach to management of their condition. In recalcitrant cases, other invasive options can be used like laser and surgical therapies [13]. One of the complications watch out for is the transformation of squamous cell carcinoma especially in patients on immunomodulators [14, 15].

## Competing interests

The authors declare no competing interests.

## References

[1] Zhao Q-F, Hasegawa T, Komiyama E, Ikeda S (2017). Hailey–Hailey disease: a review of clinical features in 26 cases with special reference to the secondary infections and their control. Dermatol Sin.

[2] Kollman N, Bass J (2018). Generalized familial benign chronic pemphigus (Hailey-Hailey disease) treated successfully with low-dose naltrexone. JAAD Case Rep.

[3] Sudbrak R, Brown J, Dobson-Stone C, Carter S, Ramser J, White J, Healy E (2000). Hailey–Hailey disease is caused by mutations in ATP2C1 encoding a novel Ca2+ pump. Hum Mol Genet.

[4] Chiaravalloti A, Payette M (2014). Hailey-Hailey disease and review of management. J Drugs Dermatol JDD.

[5] Burge SM (1992). Hailey-Hailey disease: the clinical features, response to treatment and prognosis. Br J Dermatol.

[6] Gu K, Silver S (2018). A case of Hailey-Hailey disease managed with oral magnesium citrate and high-dose Vitamin D3. J Cutan Med Surg.

[7] Xu K, Shi B, Diao Q, Jiang X, Xiao Y (2017). Identification of 2 Novel Mutations in ATP2C1 Gene in Hailey-Hailey disease and a literature review of variations in a Chinese Han population. Med Sci Monit Basic Res.

[8] Ibrahim O, Hogan SR, Vij A, Fernandez AP (2017). Low-dose Naltrexone treatment of familial benign pemphigus (Hailey-Hailey Disease). JAMA Dermatol.

[9] Kumar R, Zawar V (2008). Longitudinal leukonychia in Hailey-Hailey disease: a sign not to be missed. Dermatol Online J.

[10] LeBlanc KG, Wharton JB, Sheehan DJ (2011). Refractory Hailey-Hailey disease successfully treated with sandpaper dermabrasion. Skinmed.

[11] Starzycki Z, Chorzelski TP, Jablonska S (1998). Familial pemphigus vulgaris in mother and daughter. Int J Dermatol.

[12] Patel AA, Swerlick RA, McCall CO (2006). Azathioprine in dermatology: the past, the present, and the future. J Am Acad Dermatol.

[13] Arora H, Bray FN, Cervantes J, Falto Aizpurua LA (2016). Management of familial benign chronic pemphigus. Clin Cosmet Investig Dermatol.

[14] Holst VA, Fair KP, Wilson BB, Patterson JW (2000). Squamous cell carcinoma arising in Hailey-Hailey disease. J Am Acad Dermatol.

[15] von Felbert V, Hampl M, Talhari C, Engers R, Megahed M (2010). Squamous cell carcinoma arising from a localized vulval lesion of Hailey-Hailey disease after tacrolimus therapy. Am J Obstet Gynecol.

